# Comprehensive evaluation of nine evapotranspiration products from remote sensing, gauge upscaling and land surface model over China

**DOI:** 10.1371/journal.pone.0313762

**Published:** 2024-11-13

**Authors:** Dayang Wang, Dagang Wang, Shaobo Liu, Ya Huang, Qing Yang, Xiaohang Ma, Zequn Lin

**Affiliations:** 1 Overseas Expertise Introduction Center for Discipline Innovation of Watershed Ecological Security in the Water Source Area of the Middle Route of South-to-North Water Diversion, College of Water Resources and Modern Agriculture, Nanyang, China; 2 School of Geography and Planning, Sun Yat-sen University, Guangzhou, China; 3 State Key Laboratory of Hydrology-Water Resources and Hydraulic Engineering, Hohai University, Nanjing, China; 4 School of Freshwater Sciences, University of Wisconsin-Milwaukee, Milwaukee, Wisconsin, United States of America; KTH Royal Institute of Technology: Kungliga Tekniska Hogskolan, SWEDEN

## Abstract

Benefiting from the advancements in monitoring and measuring terrestrial evapotranspiration (ET), diverse ET products have been proliferated. This study evaluated nine ET products from three types, namely remote sensing-based retrievals (GLEAM, PML and PLSH), gauge-based upscaling (FCCRU, FCGSW and FCWFD) and land surface model-based reanalysis (ERA5-Land, GLDAS and MERRA) over China and its seven climate zones. Both spatial and temporal change trends in ET were investigated, and period feature were analyzed. Then, in-situ ET observations were used for validating the performances of ET products. The results demonstrate that all products show comparable performances in spatial distribution over China, but the mean ET values present evident discrepancies (433–563 mm/a). Among them, reanalysis ET products reproduce higher ET, but with less difference. In terms of climate sub-regions, the most significant discrepancies are located in QT. In addition, PLSH, MERRA and GLDAS present substantial increasing trends, while all three gauge-based upscaling ET products display decreasing trends. Regionally, all the ET products show positive trends in QT. Moreover, most of ET products present apparent periodic oscillation ranging from 2.0–5.5 year scales. At point scale, most ET products perform well at NMG and CBS sites (CC > 0.80, RMSE < 20 mm/month). However, general underestimations appear in northwestern China sites (HB and DX), and systematical overestimation exist in southern China sites (DHS and XSBN). By comparison, remote sensing-based ET products performs best, followed by gauge-based upscaling ET, comparatively, reanalysis-based ET products have poorest performances against in-situ ET observations. This study can provide valuable reference information for the selection of proper ET datasets for the hydrological simulation and analysis over China.

## 1. Introduction

Terrestrial evapotranspiration (ET) is an essential process interconnecting the water cycle and energy process at the interface between land and atmosphere [1, 2]. From the perspective of the global land surface, about 62% precipitation returns to the atmosphere through ET processes on average, and more than 50% of the net radiation reaching the land surface is consumed by ET. Therefore, accurate quantification of ET is crucial for many applications such as climate change, water resource management, irrigation scheduling and drought monitoring [3–5].

In-situ measurement techniques, such as pan evaporation, lysimeters, eddy covariance (EC), large aperture scintillometers, and Bowen ratio method, can directly measure ET at point and local scales [6–8]. However, sites are usually scarce, and the observed data are generally only available for a short time period, owing to the much expense of instruments. Additionally, the representativeness of site-scale observations for large scales are limited, especially in remote areas, because the coverages of land surface is usually high heterogeneous [9]. Consequently, continuing efforts have been devoted to developing large scale ET estimating in the last few decades. With the rapid development of satellite monitoring and computer techniques, ET estimates at regional, national and global scale can be achieved and a number of gridded ET datasets with different spatial-temporal resolutions have been sprung up [10, 11]. Overall, these large-scale ET datasets can be mainly divided into three categories: (1) remote sensing (RS)-based retrievals; (2) land surface model (LSM) and assimilation-based reanalysis; (3) machine learning-based upscaling of gauge observations. The remote sensing-based ET datasets are usually derived from ET models (e.g. data-driven models, energy balance models, and water-energy balance models), which require land surface variables including albedo, emissivity, land surface temperature, and vegetation-related indexes (e.g. leaf area index) observed by satellite [12–14]. LSMs can produce long-term gridded ET datasets with relative continuous and higher spatial-temporal resolution at regional to global scales [15, 16]. Meanwhile, the assimilation techniques integrate the ground-based ET observations into LSM, which can effectively help to improve the accuracy of ET simulations. Many remote sensing-based ET products and land surface model-based ET products are generated based on prescribed mechanistic and empirical models (e.g., GLEAM, PML, MODIS, Noah, VIC). Unlike them, gauge upscaling ET datasets are generated applying machine learning (or data-driven) approaches, which have been identified as a promising tool for ET estimation. Compared with the process-based and remote sensing-based models, the key characteristic of the machine learning models is that its functional relationships are not prescribed, but rather emerge from patterns found in the measurements. Hence, site upscaling ET datasets are in vogue in ET estimation studies [17, 18]. Although ET datasets are various, but there is no consensus on which ET dataset is the best, as each category has its own advantages and disadvantages. Large discrepancies and uncertainties have been evident among ET datasets, due to a consequence of different methodology principles, model assumptions, model structure deficiencies, calibration approaches, parameterization and meteorological forcing data. For example, the global averaged annual ET from 1980 to 2012 estimated by different products ranged from approximately 400 to 700 mm/a, as illustrated in previous studies [18–20]. Moreover, the ET estimates showed substantial differences, and the relative errors ranged from 14% to 44% compared with site measurements [21–23]. Furthermore, at regional scale, the differences and uncertainties seemed to be more evident [24–26]. China stretches across a vast area covering a variety of climate sub-regions and ecosystems and is facing many water issues. Accurate estimation of terrestrial ET can help to understand variability in water and energy cycles. Therefore, it is essential to evaluate the accessible gridded ET datasets prior to applying them in investigating changes in water, energy and carbon cycles in China.

In this study, nine ET products including three types, namely remote sensing-based ET products (GLEAM, PML and PLSH), gauge upscaling-based ET (FLUXCOM_CRUNCEPv8, FLUXCOM_GSWP3 and FLUXCOM_WFDEI) and land surface model reanalysis-based ET (ERA5-Land, GLDAS and MERRA) were selected to explore the discrepancies among ET datasets in different climate zones over China. Firstly, we analyzed the temporal evolutions and change trends, spatial patterns of nine ET product over various climate regions. Then, period features were calculated and investigated based on wavelet methods. In addition, we validate the ET products with in-situ ET observations from eddy covariance systems, and assessed the performances of nine ET products at point scale. This study can provide valuable and information to investigate spatial-temporal variations of different types of ET estimations in different climate zones over China.

## 2. Data and methods

### 2.1 Study area

The study area is mainland China (Taiwan were excluded for lack of data). Given the diversity of climate conditions, China can be separated into seven climate zones [27], including the Northwest desert area (NW), Inner Mongolia steppe zone (IM), Northeast humid/semi-humid warm region (NE), North China humid/semi-humid temperate zone (NC), Central and southern China humid subtropical zone (CSC), Qinghai-Tibetan Plateau (QT), and South China humid tropical zone (SC). The differences of ET among various climate sub-regions would be better compared and interpreted through this division, and it was successfully adopted employed in previous studies [28–30].

### 2.2 Data

In this study, nine gridded terrestrial ET products across China, including remote sensing-based ET products (GLEAM, PML and PLSH), upscaling-based ET products (three FLUXCOM ET datasets), and model reanalysis-based ET products (ERA5-Land, GLDAS and MERRA), were screened out and evaluated at different spatial and temporal scales. The information of these products was summarized in Table 1. Moreover, in-situ observations from eddy covariance measurements of ET were employed as ET reference to assess the nine ET products, and the site information was provided in Table 2.

**Table 1:** Summary of the nine gridded ET products employed in this study.

ET products	Types	Spatial resolution	Temporal resolution	Temporal coverage
**GLEAM**	Remote sensing-based	0.25°	Daily, Monthly	1980–2020
**PML**		0.5°	Monthly	1981–2012
**PLSH**		0.0833°	Monthly	1982–2013
**FCCRU**	Gauge upscaling-based	0.5°	Monthly	1980–2016
**FCGSW**		0.5°	Monthly	1950–2014
**FCWFD**		0.5°	Monthly	1979–2013
**ERA5L**	Reanalysis-based	0.1°	Hourly, Daily, Monthly	1950–2023
**GLDAS**		0.25°	Monthly	1945–2014
**MERRA2**		0.5°×0.625°	Monthly	1980–2020

**Table 2:** Information of flux tower sites from ChinaFLUX used in this study.

Site	Location	Elevation (m)	Ecosystem type	Data period
**Haibei (HB)**	37.62°N, 101.31°E	3250	Grassland	2003–2010
**Inner Mongolia (NMG)**	44.5°N, 117.17°E	1189	Grassland	2004–2010
**Dangxiong (DX)**	30.85°N, 91.08°E	4333	Grassland	2004–2010
**Changbaishan (CBS)**	42.4°N, 128.1°E	738	Forest	2003–2010
**Qianyanzhou (QYZ)**	26.74°N, 115.05°E	102	Forest	2003–2010
**Dinghushan (DHS)**	23.17°N, 112.57°E	300	Forest	2003–2010
**Xishuangbanna (XSBN)**	21.95°N, 101.2°E	750	Forest	2003–2010
**Yucheng (YC)**	36.95°N, 116.6°E	28	Cropland	2003–2010

#### 2.2.1 Remote sensing-based ET products

GLEAM, PML and PLSH were selected as the representations of remote sensed ET products due to these outstanding performances in estimating ET. In GLEAM model, a combination of site-based, satellite-based precipitation, radiation, air temperature data were used to calculate potential evaporation (PET) based on Priestley and Taylor equation [31]. Then, PET was converted into actual evaporation by applying a multiplicative evaporation stress factor derived from microwave observed vegetation optical depth. PML ET product was obtained based on Penman-Monteith-Leuning model, which was firstly proposed by Leuning et al. [32] and further improved by Zhang et al. [33]. The PML ET was originally from biophysical model which considers canopy physiological process and soil evaporation for the accurate estimation of surface and canopy conductance models [34]. The inputs of PML included satellite-based leaf area index, emissivity, sky shortwave albedo, reanalysis meteorological variables. PLSH ET was derived applying an improved Normalized Difference Vegetation Index (NDVI)-based Peman-Monteith algorithm developed by Zhang et al. [35]. In PLSH, ET was regulated by a set of geophysical data and vegetation index and phenology along with remote sensed radiative data and other meteorological observation reanalysis data.

#### 2.2.2 Upscaling based ET products

We selected FLUXCOM datasets as the upscaling-based ET. FLUXCOM initiative aimed to improve understanding of the multiple sources and facets of uncertainties in empirical upscaling and to provide an ensemble of machine learning-based global gridded flux products. In FLUXCOM, observations from 224 flux towers distributed over the globe were used to train machine learning models following different specifications. Additionally, it was noted that FLUXCOM database comprised 147 products in two setups of input drivers and generated global gridded products. One was using MODIS remote sensing (RS) data and the other was using remote sensing and meteorological data (RS + METEO). The former products had higher spatial resolution of 0.0833°, but they were lack of climate forcing information and had shorter span time (i.e. 2001 onwards). In contrast, the latter products made use of daily meteorological conditions and had longer span time (from ~1980 to present) depending on climate input data. However, they were limited in spatial resolution (i.e., 0.5° grid cells) subjected to the meteorological datasets. Taking into account the importance of climate condition and span time, three products of RS + METEO with different meteorological forcing datasets, including FLUXCOM_CRUNCEPv8 (1980–2016), FLUXCOM_GSWP3 (1950–2014) and FLUXCOM_WFDEI (1979–2013) were adopted in this study. Since FLUXCOM only provided latent heat flux (ET in energy units) data, it was necessary to convert the units from W/m^2^ to mm/day or mm/month using Eq (1).

ET=LE/λ (1)

where *λ* is the latent heat of vaporization assumed to be a constant of 2.45 MJ/kg.

#### 2.2.3 Reanalysis-based ET products

The reanalysis-based ET products used in this study consisted of ERA5-Land, GLDAS and MERRA. ERA5-Land was an updated land component of the fifth generation of European Reanalysis (ERA5) datasets, representing the movement of water and energy cycles over land [36]. Compared with ERA-5 and ERA-Interim, the major improvement in the ERA5-Land dataset was the higher spatial resolution of 0.1°. In addition, the improved model had dramatical updates in model structure, including improved climatological seasonality of vegetation and better parameterization for deriving bare soil evaporation [36]. GLDAS was developed jointly the National Aeronautics and Space Administration (NASA) and the National Oceanic and Atmospheric Administration (NOAA). Benefiting from ingesting satellite- and ground-based observational data products, using advanced land surface modeling and data assimilation techniques [37], GLDAS had generated optimal fields of land surface states and fluxes. Currently, GLDAS includes four LSMs: Noah, the Catchment LSM (CLSM), the Community Land Model (CLM), and the Variable Infiltration Capacity (VIC) model, of which the Noah was screened out in consideration of the data quality and temporal coverage. MERRA was produced by Global Modeling and Assimilation Office (GMAO) in NASA. It was state-of-the art reanalysis data providing long-term land surface hydrology products (e.g., soil moisture, sensible and latent heat fluxes) along with atmospheric fields. The new MERRA version 2.0 (MERRA2) was updated via integrating a wider variety of observation types from satellites and gauge sources to reproduce terrestrial ET using a water balance method [38].

#### 2.2.4 In-situ ET observations

Gauge-based eddy covariance measurements from ChinaFLUX were taken as the ET reference for validation in this study. As an important part of network AsiaFlux, ChinaFLUX was a vital observation and research network that utilized ET and chamber methods to quantify the exchanges of water vapor and energy between terrestrial ecosystem and atmosphere. Monthly observed heat flux data from 8 stations were used, and they are also converted to equivalent ET through Eq (1).

Since some products ET estimates are unavailable over the Kunlun Mountains, Taklamakan Desert and western Inner Mongolia regions, therefore, they were masked out to ensure the consistency of spatial grid cells. Moreover, due to inconsistent span time in different ET products, only the data during overlap period of 1982–2012 were extracted for evaluation across China.

### 2.3 Methodology

#### 2.3.1 Trend analysis

In this study, we applied linear fitting trend analysis, Mann-Kendall (M-K) test and Sen’s slope method to explore the change trends of different ET products. Among them, linear fitting was utilized to detect the changing trends of ET, Mann-Kendall was used to reflect the significance of the trend, and Sen’s slope was used to describe the magnitude of changes in ET time series.

The Mann-Kendall test is a nonparametric method based on the rank series, which was recommend by the World Meteorological Organization (WMO) and widely used for trend detection of hydrological variables [39, 40]. Since the autocorrelation of the original data could lead to the enhancement or decrease in the detection results to a certain extent, the pre-whitening Mann-Kendall (PW-MK) was used to remove the impact of autocorrelation in this study [41]. The equations for eliminating autocorrelation are as follows:

$$\beta =median\left(\frac{x_{j}-x_{i}}{j-1}\right),\;\forall j>i$$ (2)

$$Y_{t}=x_{t}-\beta t$$ (3)

$$r_{1}=\frac{\sum_{t=1}^{n-1}\left(x_{t}-\bar{x_{t}}\right)\left(x_{t+1}-\bar{x_{t+1}}\right)}{\sqrt{\sum_{t=1}^{n-1}{\left(x_{t}-\bar{x_{t}}\right)}^{2}\sum_{t=1}^{n-1}{\left(x_{t+1}-\bar{x_{t+1}}\right)}^{2}}}$$ (4)

$$Y_{t}^{\prime }=Y_{t}-r_{1}Y_{t-1}$$ (5)

$$Y_{t}^{\prime \prime }=Y_{t}^{\prime }+\beta t$$ (6)

Where *β* is the inclination estimator of the original time series variables based on Sen’s slope method; *Y*_*t*_ is the sequence without trend items; *r*_1_ is the first-order coefficient of autocorrelation. $Y_{t}^{\prime \prime }$ is a new sequence without an autocorrelation effect. Then, the MK trend test was applied based on $Y_{t}^{\prime \prime }$. The equations for the Mann-Kendall are expressed as:

$$S=\overset{n-1}{\underset{k=1}{\sum }}\;\overset{n}{\underset{i=k+1}{\sum }}\;Sgn\left(S_{i}-S_{k}\right)$$ (7)

$$Sgn\left(S_{i}-S_{k}\right)=\left\{\begin{array}{cc} 1 & \left(X_{i}-X_{k}\right)>0\\ 0 & \left(X_{i}-X_{k}\right)=0\\ -1 & \left(X_{i}-X_{k}\right)<0 \end{array}\right.$$ (8)

$$Var\left(S\right)=\frac{n\left(n-1\right)\left(2n+5\right)}{18}$$ (9)

$$Z=\left\{\begin{array}{cc} \frac{S-1}{\sqrt{Var\left(S\right)}} & S>0\\ 0 & S=0\\ \frac{S+1}{\sqrt{Var\left(S\right)}} & S<0 \end{array}\right.$$ (10)

Where *n* is the length of samples; *S*_*i*_ and *S*_*k*_ are sample values at times *j* and *k* respectively. *Sgn*() is a symbolic function; *S* follows a normal distribution with a mean of zero and a variance of *Var*(*S*); *Z* follows the normal distribution statistics when *n* > 10. The positive *Z* indicates an increasing trend, while negative *Z* implies a decreasing trend. Besides, the larger the absolute value means, the more significant trend. Usually, the confidence level of 95% was utilized with the |*Z*| value of 1.96.

#### 2.3.2 Period analysis

To explore the time frequency of ET, wavelet analysis was used in this study. As a new method for period analysis in time series, the wavelet presents great promise, particularly in detecting the time evolution of the parameters (period, amplitude, phase) describing periodic hydrological time series [42, 43]. The wavelet function can be defined as:

$$\int_{-\infty }^{+\infty }{\left|a\right|}^{-1/2}\varphi \left(\frac{t-b}{a}\right),\;\;a,b\in R,a\neq 0$$ (11)

$$W_{f}\left(a,b\right)={\left|a\right|}^{-1/2}\Delta t\overset{N}{\underset{k=1}{\sum }}\;f\left(k,\Delta t\right)\bar{\varphi }\left(\frac{k\Delta t-b}{a}\right)dt$$ (12)

where *φ*(⋅) represents the wavelet function; *a* denotes the wavelet scale, which can reflect the amplitude of wavelet period; *b* is localized time index, indicating how this amplitude varies with time. *W*_*f*_(*a*, *b*) is the wavelet transform, *k*Δ*t* denotes the discrete of *t*; $\bar{\varphi }\left(\cdot \right)$ represents the conjugate function of *φ*(⋅). Then, the wavelet variance can be derived by integrating the square values of the wavelet coefficients, as follows:

$$Var\left(a\right)=\int_{-\infty }^{+\infty }{\left|W_{f}\left(a,b\right)\right|}^{2}\;db$$ (13)

where *Var*(⋅) denotes the wavelet variance, which varies with the wavelet scale and reflect the distribution of signal energy fluctuation with scales. The main period of time series can be captured based on the fluctuation of energy.

#### 2.3.3 Accuracy evaluation metrics

The nine ET products were compared with the in-situ observational ET. Various statistical metrics, including root mean square error (RMSE) and correlation coefficient (CC) were used to evaluate the performances of ET products at point scale.

$$\text{RMSE}=\sqrt{\frac{1}{N}\overset{N}{\underset{i=1}{\sum }}{\left(S_{i}-R_{i}\right)}^{2}}$$ (14)

$$\text{CC}=\frac{\sum_{i=1}^{N}\left(S_{i}-\bar{S}\right)\left(R_{i}-\bar{R}\right)}{\sqrt{\sum_{i=1}^{N}{\left(S_{i}-\bar{S}\right)}^{2}\sum_{i=1}^{i=N}{\left(R_{i}-\bar{R}\right)}^{2}}}$$ (15)

where *N* indicates the total number of months; *S*_*i*_ and *R*_*i*_ denote the ET products values and site-based ET values, respectively; the subscript *i* represents *i*th months. $\bar{S}$ and $\bar{R}$ represent the temporal averaged ET from products and observations.

## 3. Results

In this section, nine ET products mentioned above were evaluated based on seven climate zones from spatial and temporal perspectives and validated with in-site observed ET data at point scale. Due to the spatial resolution mismatch among ET products, all of which were resampled to the same spatial resolution of 0.5×0.5 grid cells and aggregated to monthly scale for the overlap period of 1982–2012. For GLEAM, PLSH, ERA5L and GLDAS ET products, we upscaled them to 0.5 degrees grid cells via arithmetic average because of the higher resolution. As for MERRA, which was resampled by applying bilinear interpolation. Moreover, it should be noted that parts of pixels ET located in northwestern China were missing values, therefore they were masked out from all of the ET products for consistency. For clearly distinguishing, hereafter, FLUXCOM_CRUNCEPv8, FLUXCOM_GSWP3 and FLUXCOM_WFDEI were denoted as FCCRU, FCGSW and FCWFD, respectively, ERA5-Land were denoted as ERA5L.

### 3.1 Spatial distribution of ET products

**Fig 1:**
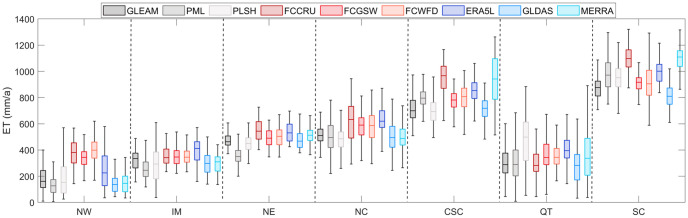
Boxplot of multiyear averaged ET during 1982–2012 in the seven climate zones of China.

Overall, the nine products consistently present southeast to northwest decreasing trend in the mean annual ET over China. The long-term averaged values of ET products range from 433 mm/a (from GLDAS) to 563 mm/a (from FCCRU) during the period of 1982–2012, demonstrating the significant discrepancies in amount of annual mean ET. The ET high-value located in southeastern China can reach above 900 mm/a, while the ET low-value distributed in northwestern China is only below 100 mm/a. As for climate regions, the boxplot of ET averaged values is displayed with boxplot in Fig 1. It can be seen that ET values show significantly diverse performances in different climate sub-regions. Of them, CSC and SC zones have much higher values statistical indicators (i.e., median value, maximum value and minimum value) of ET, owing to the higher temperature and more precipitation. Moreover, the ranges among different ET products are also inconsistent. In contrast, the inconsistencies are relatively lower in IM and NE, and most of ET products have comparable ET median. However, the upscaling ET products present higher ET values than other ET products, while remote sensing-based ET products perform lower ET median over most of climate regions than other types of products. It is worth noting that wider ET range can be seen over Qinghai-Tibetan Plateau (QT) in almost all of ET products, although with lower ET average values (Fig 1). It is probably attributed to the diversity vegetation coverage and various atmospheric conditions in QT, where the ET processes could be more complex and changeable.

### 3.2 Temporal evolution of ET products

**Fig 2:**
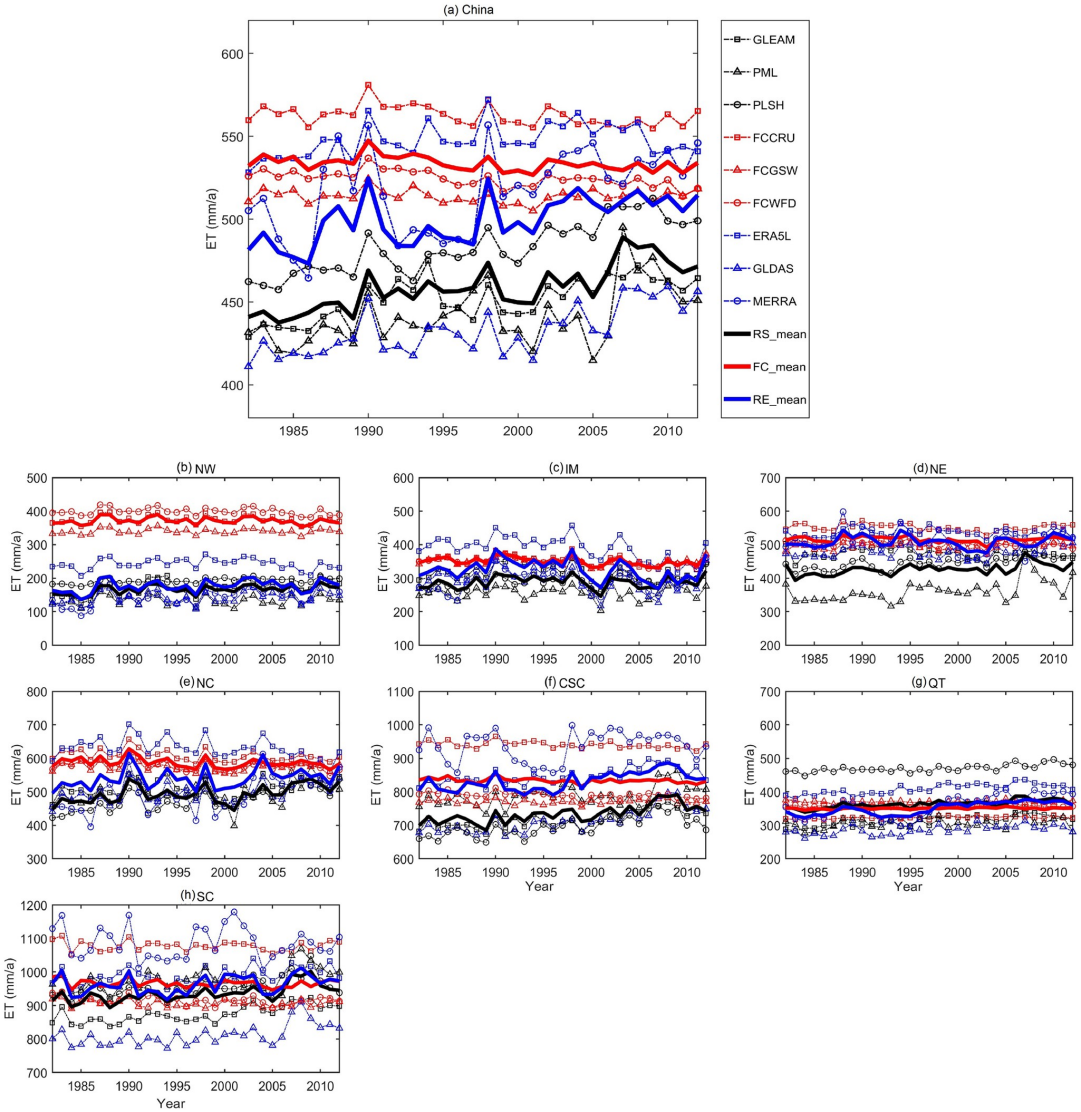
Annual mean time series of ET in China and seven climate zones from 1982 to 2012. (Thin dashed lines with different color represent the different types of ET products, black represents remote sensing-based ET, red denotes gauge upscaling-based ET and blue indicates reanalysis-based ET. Thick solid lines represent the average values of the same types of ET products).

To investigate the discrepancies among different types of ET estimates, the evolution of annual time series of nine products over China and its seven climate sub-regions are depicted in Fig 2. From the perspective of the whole China, annual ET varies from approximately 400 mm/a to 550 mm/a with similar fluctuation patterns for the period of 1982–2012. However, noticeable differences still exist among the three types of ET products. For instance, gauge upscaling-based ET products (i.e., FCCRU, FCGSW, and FCWFD) present generally higher ET values with almost all of the annual ET values over 500 mm/a. By comparison, remote sensing-based ET products show lower ET values with mean ET values approximately 450 mm/a. Despite relatively moderate ET amounts, the discrepancies among the three reanalysis-based ET products (i.e., GLEAM, PML, and PLSH) are the most obvious. As for climate regions, the differences remarkably vary with zones. For example, all three types of ET products have closer average values in QT, while they exhibit distinct discrepancies in NW (Fig 2b and 2g). The mismatches and inconsistencies among different types of ET maybe partially stemming from various climate conditions over NW, where ET processes are highly susceptible and complex.

**Fig 3:**
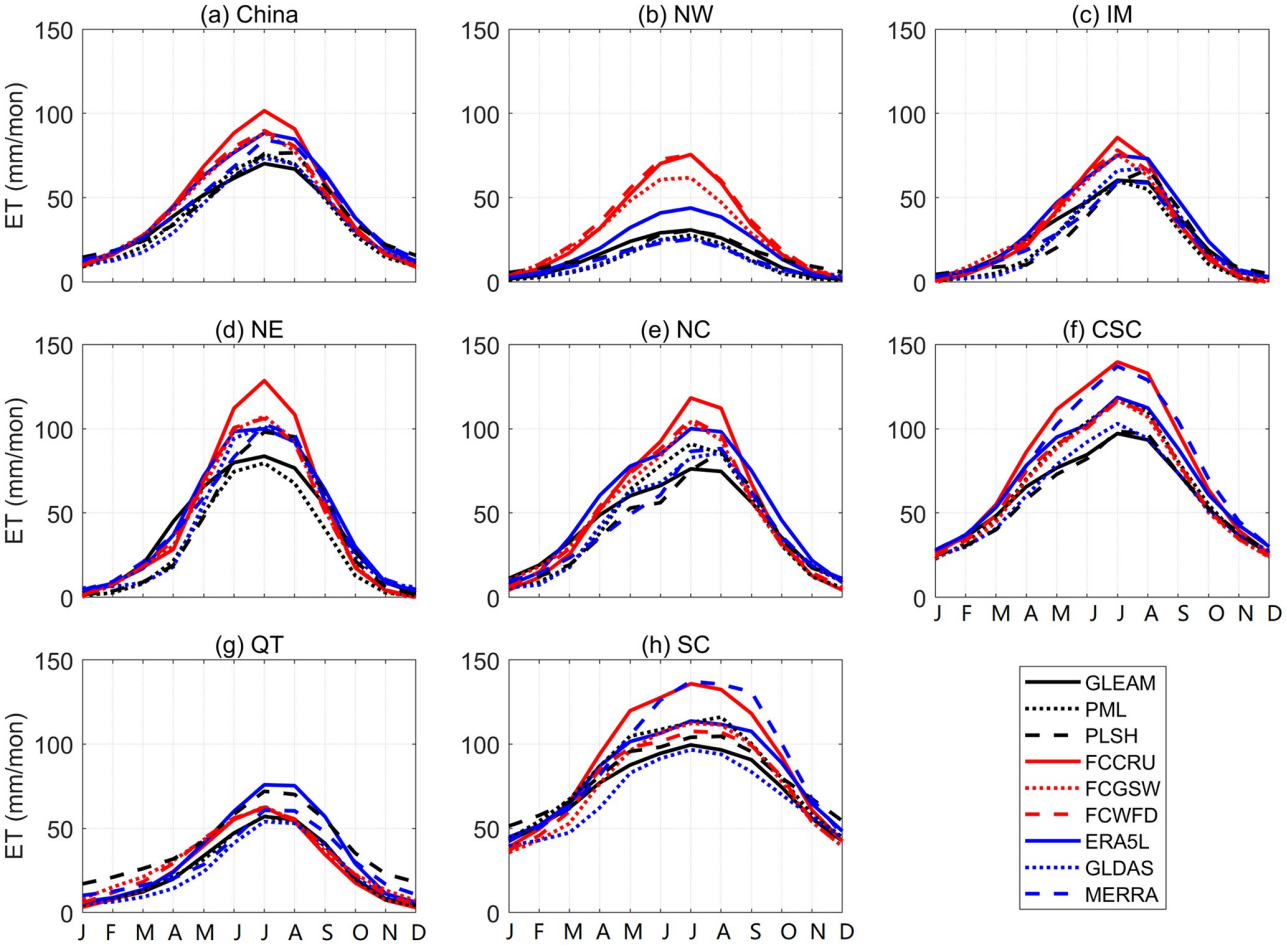
Seasonal cycles of nine ET products over China and its seven climate sub-regions during the period of 1982–2012.

Furthermore, we analyzed climatological seasonal cycles of nine ET products over China and its seven climate zones during 1982–2012, as shown in Fig 3. Generally, nine ET products show consistent seasonal fluctuations, with high values in the warm season (June-July-August) and lower values in the cool season (December-January-February). In terms of climate sub-regions, the seasonal cycles can be classified into two types according to the patterns. One can be called “thin-high”, which includes NE, NC, CSC and SC. The other can be depicted “heavy-low”, which includes NW, IM and QT. Moreover, the distinct differences described above in NW among different ET products are mainly attributed to the warm season. In summer, the ET values of FCCRU and FCWFD can reach 75 mm/month, which are almost three times higher than that of PML and PLSH (about 25 mm/month). By comparison, the ET values of different products are closer in cool season.

### 3.3 Trend analysis of ET products

**Fig 4:**
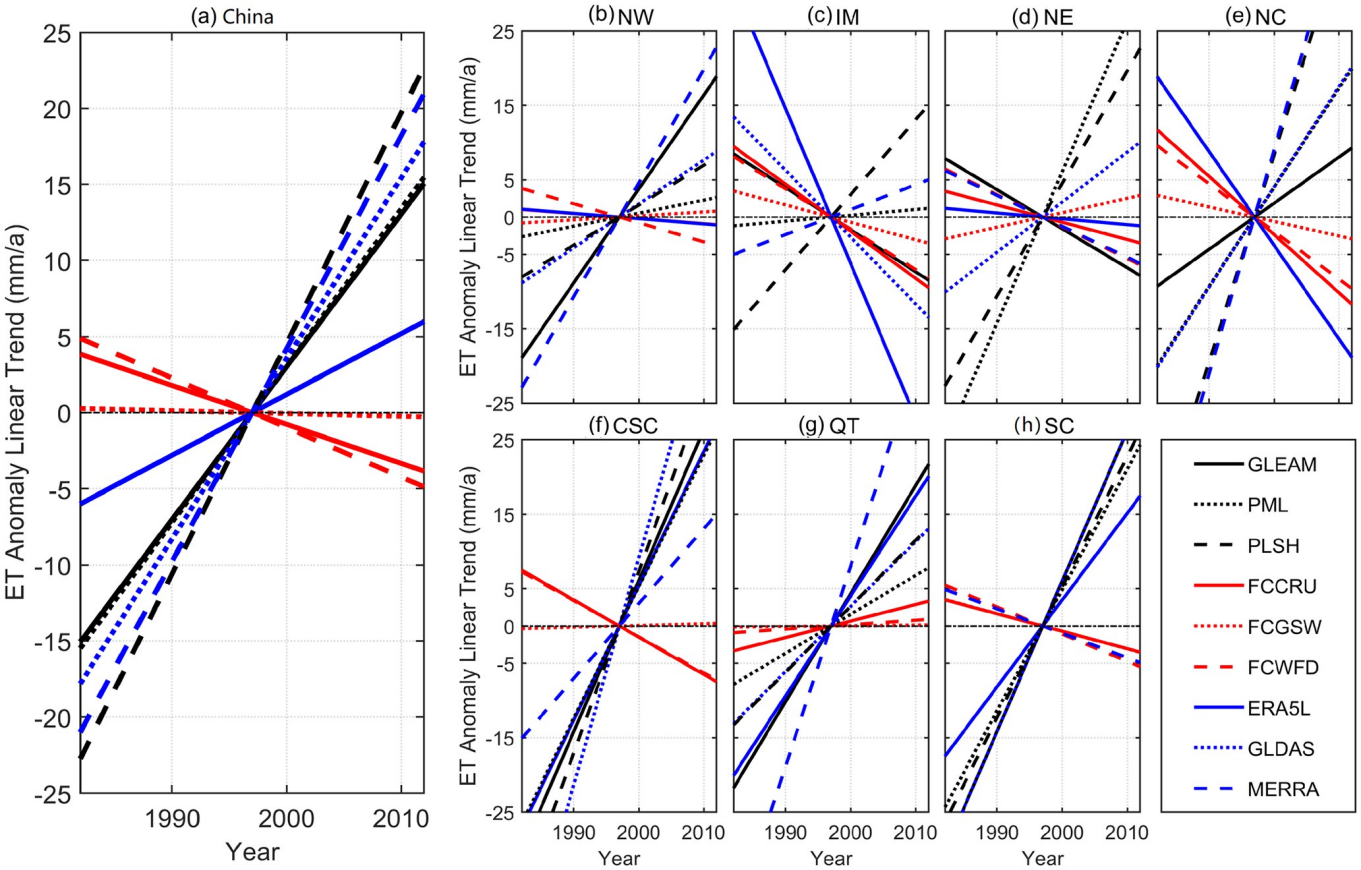
Temporal change trends of ET anomalies over China and its seven climate sub-regions during 1982–2012. (Only the linear-fittings are retained, and the same color indicates the same type of ET products, of which blue black represents remote sensing-based ET, red denotes gauge upscaling-based ET and blue is reanalysis-based ET).

In this study, we analyzed the change trends of nine ET products. Given that there may be systematical errors, ET anomalies instead of ET values were used to explore the change trends during the period from 1982 to 2012. The temporal and spatial change trends were provided in Fig 4, respectively. Overall, both remote sensing-based and reanalysis-based ET products show significant increasing trends. Of these, the largest increasing rate can be found in PLSH (1.52 mm/a), followed by MERRA (1.40 mm/a) and GLDAS (1.19 mm/a). Comparatively, all the three gauge upscaling-based ET products present decreasing trends, with rates of -0.256 mm/a (FCCRU), -0.018 mm/a (FCGSW) and -0.326 (FCWFD). It implies that the ET trends are substantially different, due to the differences among methodology principles. Furthermore, these differences can also be observed in most of the climate sub-regions. However, it should be highlighted that all of the ET products show positive trends in QT (Fig 4g). Specifically, there is a drastic increasing in the middle of the QT sub-region, with a rate reaching 3.0 mm/a. In addition, most of ET grids in CSC also present evident increasing trends, because of the greening vegetation cover and warming temperature. Notably, all the magnitudes of change rates in the three gauge upscaling-based ET products are less than 1.0 mm/a. The reason may be explained by the fact that FLUXCOM products are the averaged results of various machine learning algorithms. Moreover, in IM climate zone, approximate half of ET products display negative trends, with a decreasing rate of exceeding -4.0 mm/a in some areas.

### 3.4 Period analysis of ET products

**Fig 5:**
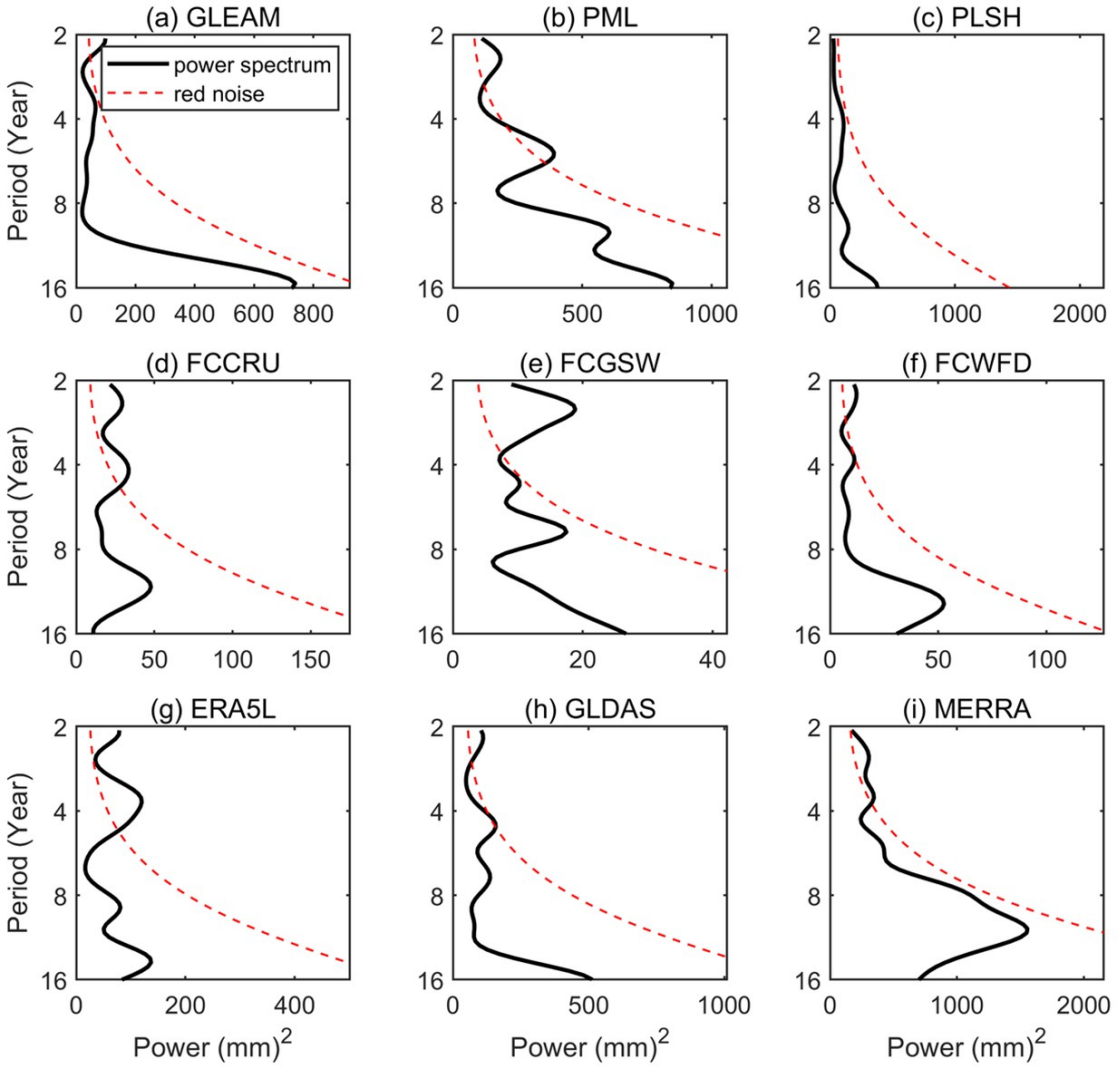
Wavelet power spectrum of annual averaged ET from nine ET products over China during the period of 1982–2012. (The red dash line represents the 95% confidence level for a red noise null hypothesis. If the peak value of entire black solid line exceeds the red imaginary line, which suggests that the corresponding period is significant).

The time frequency features of nine ET products were explored based on wavelet power spectrum method, as displayed in Fig 5. It can be seen that most of ET products have apparent periodic oscillations ranging from 2.0 year to 5.5 years scales during the period 1982–2012 varying with products. For instance, there are domain periodic oscillations of 2.4 year and 5.1 year at the 95% confidence level within PML during 1995–2010. In FCCRU, inter-annual oscillations at 2.4–4.1 year scales are evident from 1995 to 2010. The main periodic oscillations in ERA5L, GLDAS and MEERA are 3.6 year, 4.4 year and 3.5 year, respectively. Moreover, a longer periodic oscillations of 10.5 year can also be discovered in MERRA during the period of 1982–1995, although it is not at the 95% confidence level.

### 3.5 Grid-site evaluation

**Fig 6:**
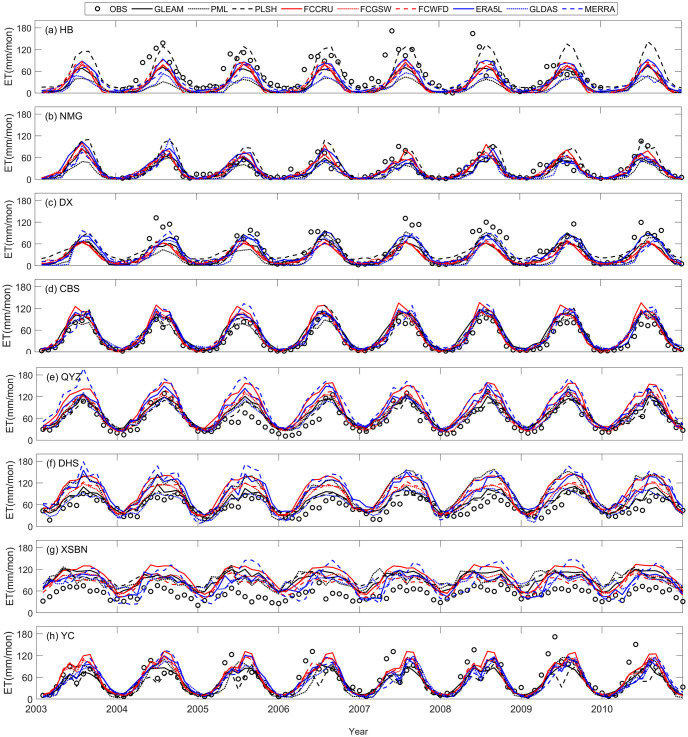
Time series of monthly ET estimates from the nine ET products and site-based ET observations at the 8 stations from ChinaFLUX.

**Fig 7:**
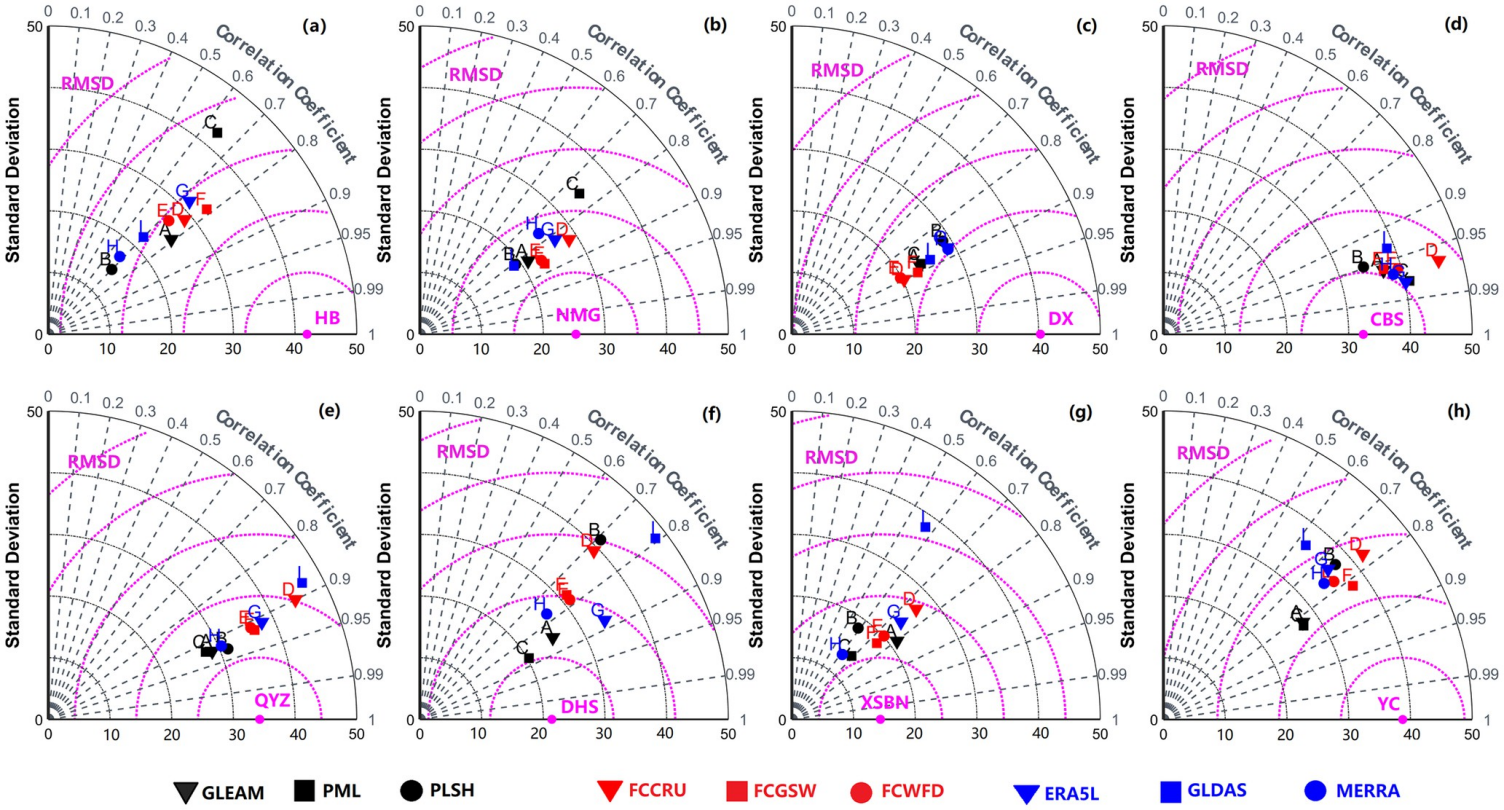
Taylor diagram of nine ET products with flux tower observations as the reference at eight stations. (The makers with the same color represent the same types of ET products, and makers labeled by A-I indicate GLEAM, PML, PLSH, FCCRU, FCGSW, FCWFD, ERA5L, GLDAS and MERRA, respectively).

In order to assess the performances of different ET products, we compared them with in situ observed ET data. The grid cell containing ChinaFLUX tower was screened out for evaluation according to the geographic coordinate information of stations. Inevitably, there may be some uncertainties when directly comparing grid-based ET with point-based ET, due to the relatively limited footprint of flux sites. However, this method can still provide valuable reference results about the performances of nine ET products at local scale, because in situ ET observation was always taken as the truth of ET. Fig 6 illustrates the comparison of monthly ET estimates from the nine ET products at eight flux sites from ChinaFLUX during the period of 2003–2010. The taylor diagrams including standard deviation (SD), RMSE and CC are provided in Fig 7. As a whole, all nine ET estimates capture the fluctuation patterns of monthly averaged ET at all sites, especially at NMG and CBS (Fig 6d and 6e). The RMSE values at these sites are less than 20 mm/month, and CC values are greater than 0.80 (Fig 7d and 7e). However, there are still apparent discrepancies among ET products in some sites. For instance, most of ET products tend to perform underestimation against observed ET at HB (almost in entire year) and DX (especially in summer), except for PLSH. The largest RMSE value of 53.3 mm/month comes from PML, when compared with gauge ET observation at HB. Meanwhile, the corresponding CC value is only 0.64. What’s more, the other remote sensing-based ET products (i.e., GLEAM and PLSH) also present undesirable performance at HB site, which implies more efforts need to be made to satellite-based monitoring techniques and ET retrieval models in northwest China. Conversely, DHS and XSBN, located in south China humid tropical zone, all the ET products show overestimation when compared with in-situ ET measurement. The greatest RMSE value of 51.9 mm/month results from FCCRU, and the smallest CC value of 0.57 was derived from MERRA at XSBN. On average, remote sensing-based ET products outperform than the other two types of ET products, with smallest RMSE value of 26.9 mm/month, followed by gauge-based upscaling ET products (28.5 mm/month). Oppositely, the reanalysis-based ET products have poorest performances at point scale.

## 4. Discussion

This study systematically assessed the performances of nine ET products from three types of ET estimation based on multiple temporal and spatial scales. Although a number of valuable results have been obtained, several issues are still worth discussing.

Firstly, parts of areas in northwest China were masked out due to the missing values in several ET products. It is probably attributed to the complex and changeable climate condition, and the unique ecosystems are extremely fragile and sensitive to climate change and vegetation dynamics [44, 45]. Therefore, most of ET algorithms seem incapable of accurately estimating ET over northwest China, especially in arid and semi-arid areas. Wang et al. [46] conducted a series of hydrological simulation applying land surface model with different meteorological dataset to explore the impact of atmospheric forcing on ET processes. The results demonstrated that remarkable biases (either significant overestimations or underestimations) exist over northwestern China, especially in northern QT and southern and eastern NW. Different from other terrestrial regions, due to the steep topography, complex landforms, harsh and uninhabitable climate, in-situ observation records on QT are relatively scare. Furthermore, the exchange of water vapor between land and atmosphere may be more intensive. Therefore, it is a challenging task for both remote sensing-based model and land surface model to estimate accurately ET measurements. Therefore, there were remarkable discrepancies existed among different ET products. Moreover, northern China has experienced large-scale soil erosion, soil degradation, and desertification over the last several decades, which furtherly complicate terrestrial process [47].

Secondly, gauge upscaling-based ET estimates do not present very outstanding performances, although they were reproduced by taking site-based ET measurements as the targets for training and calibration based on various machine learning models and algorithms. The reason may be due to the lack of in-situ long-term ET observation over China, which impedes the validation and limits the function of model to some extent [48]. Additionally, different machine learning models are usually based on diverse learning approaches and mathematical algorithms (e.g., supervised machine learning, unsupervised machine learning, semi-supervised machine learning and reinforcement learning), which all have their own benefits and potential drawbacks. Yin et al. [49] employed six machine learning models to improve the terrestrial ET estimation and evaluated the effectiveness of different algorithms at site scale and basin scale. The results showed that large uncertainties and biases still remain because of the algorithms principle, atmospheric effects, the consecutiveness of land surface condition (e.g. NDVI and LAI), insufficient representative land use and coverage, and so on.

Thirdly, differences still exist even within the same type of ET products. For example, the remote sensing-based ET products, such as GLEAM and PLSH, both are used in this study. However, the former algorithm was based on modified Penman–Monteith equation, whereas the latter applied modified Priestley–Taylor equation. Besides, the former strongly depended on the net radiation data than vapor pressure deficit (VPD) and surface and aerodynamic resistances. The latter energetically required high quality meteorological forcing data. In terms of land surface model-based ET, most models consider the physiological effect of CO_2_ on stomatal closure, when comparing with remote sensing-based ET products. However, most models typically do not allow integration of observation-based vegetation characteristics, which caused the uncertain among different models.

Finally, according to the evaluated results, almost all the ET products overestimate ET values in XSBN, which was also reported in previous studies. Sun et al. [50] found that both CLSM-based and Noah-based simulated ET presented systematic positive biases at XSBN sites. Wang et al. [46] simulated ET with three meteorological datasets and obtained the similar overestimations. These biases may be partially explained by the specially underlying surface conditions. XSBN is located in tropical forest with dense and stacked vegetation, thus reasonable LAI measurement may be challenging. Consequently, neither land surface model-based nor remote-sensing may fail to accurately capture dynamic variation of vegetations. Besides, owing to the thick forest in XSBN, shaded leaves are not light-saturated, which would lead to diffuse sunlight condition and a higher fraction of photosynthetically active radiation in the shaded leave areas [51, 52]. Then, these may result in the substantial biases of land surface model-based ET simulations as well as satellite-based ET retrievals.

## 5. Conclusions

This study evaluated nine ET products, including remote sensing-based ET products (GLEAM, PML and PLSH), gauge upscaling-based ET products (FCCRU, FCGSW and FCWFD), and land surface model reanalysis-based ET products (ERA5-Land, GLDAS and MERRA) over China for a time span of 31 years (1982–2012). The change trends of ET were analyzed from multiple temporal and spatial scales using linear fitting trend analysis, Mann-Kendall test, and Sen’s slope method from the perspectives of the seven climate zones located in China. Besides, the time frequency features of ET products were also investigated applying wavelet power spectrum method, and in-situ ET observations from ChinaFLUX were used to validate the performances of these products at point scale. The main conclusions are summarized as follows:

All ET products reproduce comparable spatial patterns over China, while multiyear averaged ET values show apparent discrepancies, ranging from 433 mm/a and 563 mm/a. Among them, gauge upscaling-based ET products show higher ET products, followed by reanalysis-based ET products. At climate sub-regions, most of ET products present close ET median over the same zones, but the ranges between maximum value and minimum values of ET are distinct, especially in QT. As for seasonal fluctuations, NE, NC, CSC and SC show “thin-high” patterns, whereas NW, IM and QT display “thin-high” patterns. In terms of change trends, the most significant increasing trends can be found in PLSH (1.52 m/a), followed by MERRA (1.40 mm/a) and GLDAS (1.19 mm/a), while three gauge-based upscaling ET products have decreasing trends, such as FCCRU (-0.256 mm/a), FCGSW (-0.018 mm/a) and FCWFD (-0.326). Regionally, all the ET products show positive trends in QT, and half of products display negative trends in IM, with a decreasing rate of exceeding -4.0 mm/a. Moreover, most of ET products present apparent periodic oscillations ranging from 2.0–5.5 year scales during the evaluation period, but a longer periodic oscillation of 10.5 year within MERRA. As for validation analysis at point scale, all ET products perform well in capturing the fluctuation patterns of monthly evolution, especially at NMG and CBS, with RMSE values of less than 20 mm/month and CC values of larger than 0.80. At the sites located in northwestern China (i.e., HB and DX), most of ET products tend to underestimate ET against observed ET. In contrast, At the sites located in southern China (i.e., DHS and XSBN), all the ET products present substantial overestimation when compared with in-situ ET measurements. In short, remote sensing-based ET products performs best, followed by gauge upscaling-based ET, comparatively, reanalysis-based ET products have poorest performances at point scale.
